# Improving Risk Stratification in Sudden Cardiac Death Using Interpretable Machine Learning: A Clinical Perspective

**DOI:** 10.3390/healthcare13212788

**Published:** 2025-11-03

**Authors:** Hana Ivandic, Branimir Pervan, Vedran Velagic, Alan Jovic, Mislav Puljevic

**Affiliations:** 1 Faculty of Electrical Engineering and Computing, University of Zagreb, Unska 3, 10000 Zagreb, Croatia; hana.ivandic@fer.unizg.hr (H.I.); branimir.pervan@fer.unizg.hr (B.P.); alan.jovic@fer.unizg.hr (A.J.); 2 University Hosiptal Centre Zagreb, Ulica Mije Kišpatića 12, 10000 Zagreb, Croatia; vedran.velagic@kbc-zagreb.hr; 3 School of Medicine, University of Zagreb, Šalata 2, 10000 Zagreb, Croatia

**Keywords:** sudden cardiac death, implantable cardioverter-defibrillator, interpretability, machine learning, logistic regression

## Abstract

**Background**: Sudden cardiac death (SCD) remains a major cause of cardiovascular mortality. Implantable cardioverter-defibrillators (ICDs) reduce arrhythmic mortality, but current selection based largely on left ventricular ejection fraction (LVEF) lacks precision. Many patients undergo device implantation without ever receiving therapy, while others at risk remain unprotected. Interpretable machine learning (ML) can integrate diverse clinical variables and refine patient selection while maintaining transparency in clinical reasoning. **Methods**: We retrospectively analyzed 607 patients who underwent ICD or CRT-D implantation at a Croatian tertiary care center. Baseline demographic, clinical, echocardiographic, laboratory, and device-related variables were collected. Patients were followed through routine device interrogations, with appropriate ICD activation serving as a surrogate for SCD prevention. A logistic regression (LR) model was trained to predict appropriate device activation. **Results**: LR model demonstrated strong predictive ability (AUC-ROC 0.74, sensitivity 86.50%). Significant predictors included ventricular tachycardia (VT) burden, sustained VT, longer follow-up, and secondary prevention. The combination of furosemide and spironolactone therapy was linked to lower predicted SCD risk. **Conclusions**: ML applied to routinely collected data can support risk stratification in SCD and complement existing guideline criteria by reinforcing known predictors and uncovering novel associations.

## 1. Introduction

Sudden cardiac death (SCD) remains one of the leading causes of cardiovascular mortality worldwide, responsible for many deaths each year despite advances in pharmacological and interventional therapies. Implantable cardioverter-defibrillators (ICDs) are proven to reduce arrhythmic mortality in patients with ischemic cardiomyopathy and impaired ejection fraction, as established by landmark trials such as MADIT, MUSTT, and SCD-HeFT [1,2,3]. However, in non-ischemic cardiomyopathy, benefits are less consistent. The DEFINITE trial showed a trend toward mortality reduction that did not reach statistical significance [4], while the DANISH trial demonstrated no overall survival benefit, except in younger patients (<70 years) who appeared to derive better protection from SCD with ICDs [5,6]. Subsequent meta-analyses confirmed that the benefit in non-ischemic populations is modest and heavily dependent on individual patient factors [7].

These observations highlight a fundamental challenge. Current risk stratification criteria, particularly reliance on left ventricular ejection fraction (LVEF), lack sufficient precision. A considerable proportion of patients with severely reduced LVEF never experience malignant ventricular arrhythmias or ICD therapies, while others with milder functional impairment remain at risk [8,9,10]. Additional markers, such as myocardial fibrosis identified via cardiac magnetic resonance imaging (CMR), can refine risk assessment, yet are not universally available and still do not fully resolve the problem of patient selection [11,12,13]. Thus, clinicians are confronted with a dilemma of implanting devices broadly and risking unnecessary procedures, or restricting indications and missing patients who could have been protected.

Clinical data from real-world cohorts reinforce this issue. At University Hospital Centre Zagreb, a large proportion of patients received ICDs in accordance with guideline criteria but never required appropriate therapy, thereby undergoing an invasive procedure with procedural and long-term risks without tangible benefit. Conversely, among those who did receive appropriate therapy, device intervention was lifesaving in the majority of cases. These contrasting outcomes underscore the urgent need for more precise tools to identify patients truly at risk of SCD and those unlikely to benefit from device implantation.

In this context, artificial intelligence (AI) offers an appealing solution. Unlike conventional approaches, which rely on a limited set of predictors, machine learning (ML) models can integrate heterogeneous data sources, ranging from demographics and comorbidities to device parameters and pharmacotherapy, and uncover complex, non-linear patterns. Systematic reviews have reported that across different populations and methodologies, ML models achieve area under the receiver operating characteristic curve (AUC-ROC) median values in the range of 0.76 to 0.81 for cardiac resynchronization therapy prediction [14], SCD prediction [15], and prediction of malignant ventricular arrhythmias treated by the ICD [16]. Some multimodal approaches, combining electrocardiographic and clinical variables, have even reported performance up to 0.90 [17]. Models trained on ECG or imaging features have shown encouraging results, with predictive value maintained even when data were collected months before an SCD event [18,19]. Furthermore, AI has also been applied to predict inappropriate ICD shocks, hospitalizations, and all-cause mortality in ICD populations, extending its potential beyond simple arrhythmic risk estimation [20,21].

The Croatian ICD cohort [22] offers a valuable framework for evaluating such methods in practice. It includes both ischemic and non-ischemic patients, treated according to prevailing European Society of Cardiology (ESC) guidelines [10], with systematically collected clinical and device data over long-term follow-up. Leveraging this dataset, we investigated whether the logistic regression (LR) model could identify patients most likely to experience appropriate ICD activation, which serves as a surrogate for SCD prevention. The emphasis of this study is not the technical analysis itself, but rather the clinical implications of applying such methods.

The overarching goal of this work is therefore to examine whether routinely available clinical data, analyzed with AI techniques, can improve upon traditional criteria for SCD risk stratification defined by currently valid ESC recommendations [10]. By focusing on appropriate ICD activation as a clinically meaningful endpoint, we sought to explore whether AI could complement guideline-based decision-making, reduce unnecessary device implantations, and ensure that high-risk patients are reliably protected. This perspective is particularly relevant as European guidelines have already acknowledged the shortcomings of an LVEF-driven approach and called for more individualized strategies [10]. While AI has not yet been incorporated into formal clinical guidelines, it is increasingly recognized as part of the future landscape of arrhythmic risk assessment [23].

## 2. Materials and Methods

This was a retrospective, single-center observational study conducted at the University Hospital Centre Zagreb, the largest tertiary referral institution in Croatia. The study population included 614 consecutive patients who underwent implantation of an implantable cardioverter-defibrillator (ICD) or cardiac resynchronization therapy with defibrillator (CRT-D) between January 2010 and December 2018. All implantations were performed according to the ESC guidelines valid at the time, which recommended ICD therapy for patients with ischemic or non-ischemic cardiomyopathy and left ventricular ejection fraction (LVEF) ≤ 35% despite optimal medical therapy, or for secondary prevention after documented ventricular tachyarrhythmias or resuscitated cardiac arrest [10]. Appendix A gives an overview of all variables used and their distribution.

### 2.1. Data Collection

Clinical and device-related data were obtained retrospectively from electronic health records, implant registries, and device interrogation reports. Baseline demographic variables included age and sex. Clinical data encompassed etiology of cardiomyopathy (ischemic vs. non-ischemic), New York Heart Association (NYHA) functional class, and a detailed profile of comorbidities, including arterial hypertension, diabetes mellitus, chronic kidney disease, atrial fibrillation, history of stroke or transient ischemic attack, peripheral artery disease, chronic obstructive pulmonary disease, and history of malignancy.

Medication use at baseline was systematically recorded, focusing on guideline-directed heart failure therapies: angiotensin-converting enzyme inhibitors (ACEI) or angiotensin receptor blockers (ARB), beta-blockers, mineralocorticoid receptor antagonists (MRA), diuretics, and, in later years, angiotensin receptor–neprilysin inhibitors (ARNI). Device type (single-chamber ICD, dual-chamber ICD, or CRT-D) and implantation indication (primary vs. secondary prevention) were documented.

Echocardiographic data at the time of implantation included left ventricular ejection fraction, left ventricular end-diastolic diameter, and the presence of significant valvular disease. Laboratory data included renal function (serum creatinine and estimated glomerular filtration rate), serum electrolytes, hemoglobin level, and natriuretic peptides, where available.

### 2.2. Device Follow-Up and Outcomes

Patients were followed through routine outpatient visits and device interrogations. Device implantations and routine follow-up were performed by the co-authors (V. Velagic and M. Puljevic) together with their cardiology team, in direct clinical interaction with the patients. For this study, all variables and outcomes were abstracted retrospectively from existing electronic health records, implant registries, and device interrogation reports, i.e. no study-specific patient contact was undertaken.

All device activations were reviewed and categorized as appropriate or inappropriate. Appropriate ICD therapy was defined as antitachycardia pacing or shock delivered in response to documented sustained ventricular tachycardia (VT) or ventricular fibrillation (VF). Inappropriate therapy was defined as device activation triggered by supraventricular tachyarrhythmias, atrial fibrillation, sinus tachycardia, or oversensing.

Patients were classified according to whether they experienced appropriate device therapy during follow-up, thereby identifying those who would likely have suffered sudden cardiac death in the absence of device intervention. Those without appropriate device therapy were considered to have undergone implantation without demonstrable survival benefit, despite being guideline-eligible.

### 2.3. AI-Based Analysis

To explore whether AI could improve risk stratification, routinely available clinical, laboratory, and device-related data were analyzed using LR. Although more advanced ML techniques have been utilized in a similar context, we opted for LR for multiple reasons. Firstly, LR, as one of the de facto standard ML methods, is inherently interpretable, which is an aspect of paramount importance in clinical settings. Secondly, the data used in the study is plainly tabular, which eliminates the necessity for employing more complex ML methods and opens the space to optimize for computational efficiency. Finally, we aimed to illustrate that even a relatively simple model can yield robust results. The model was optimized to prioritize sensitivity in detecting patients who experienced appropriate ICD therapy, reflecting the clinical imperative to avoid missing high-risk individuals. The model was trained on 80% of the dataset, and the remaining 20% was used to evaluate its performance. Model optimization was performed using 10-fold cross-validation with grid search, and the confidence interval was determined using bootstrapping. The variables were encoded as numerical values and standardized, and missing data were imputed with 0. To address the dataset imbalance, undersampling was applied to the training set.

The methodological focus of this study was deliberately clinical rather than technical. Therefore, detailed algorithmic parameters and computational pipelines are not reported here, as they are outside the scope of this paper. Instead, the emphasis is placed on the translational value of AI applications in clinical practice and on the insights these models provide into patient risk stratification.

### 2.4. Ethics

The study was conducted in accordance with the Declaration of Helsinki and approved by the institutional ethics committees of both the University Hospital Centre Zagreb and the University of Zagreb Faculty of Electrical Engineering and Computing. Given the retrospective design, no informed consent was required.

## 3. Results

A total of 607 patients underwent ICD or CRT-D implantation during the study period and were included in the final analysis. The original dataset included 614 patients, but seven were excluded from the study due to a short post-implantation follow-up of less than one month. The mean age was 58.7 years, with a strong predominance of men (83%). The majority of patients presented with reduced LVEF (mean 31.5%) and belonged to NYHA functional classes II or III at the time of implantation. Slightly more than half of the cohort had non-ischemic cardiomyopathy, while the remainder had ischemic cardiomyopathy.

During a median follow-up of 46 months, device activation occurred in 31.3% of patients. In 88% of these cases, the activation was appropriate, corresponding to successful termination of ventricular tachycardia or ventricular fibrillation. Thus, approximately one in three patients benefited directly from ICD intervention, whereas the majority of patients never experienced a device activation despite guideline-based implantation. This imbalance highlights the persistent problem of imprecise patient selection.

### 3.1. Performance of the AI Model

The LR model demonstrated promising predictive ability when applied to routinely available clinical, echocardiographic, laboratory, and device-related variables. It achieved a sensitivity of 86.5%, an accuracy of 64.8%, and a precision of 45.7%. This indicates that the model correctly identified a high percentage of all patients who ultimately required life-saving ICD therapy. Although precision was lower, leading to a higher proportion of false positives, this trade-off was considered acceptable in the context of clinical decision-making where missing high-risk individuals is the greatest concern. The confusion matrix is available in the Appendix B.

**Figure 1:**
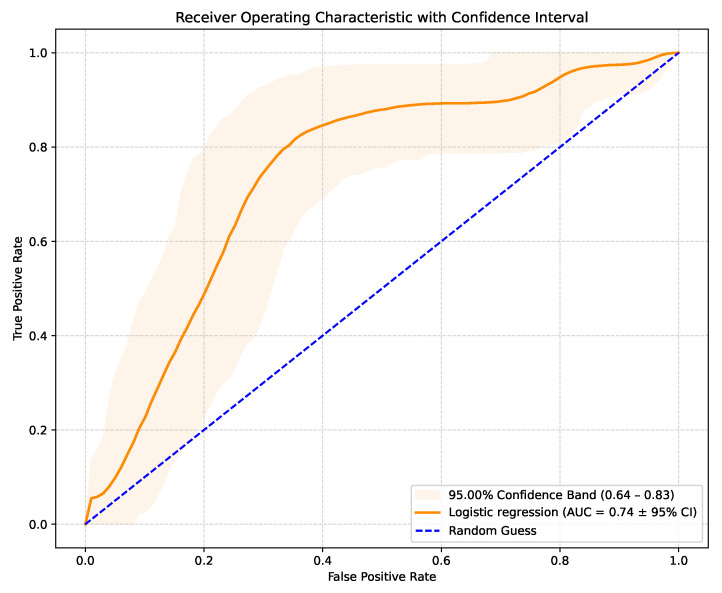
AUC-ROC curve with confidence interval.

The LR model produces raw probabilities of an SCD occurring in a specific patient. By varying the classification threshold from 0 to 1, true positive and false positive rates can be computed at each threshold, generating the ROC curve. This allows the evaluation of the model’s discriminative ability. The model achieved an AUC-ROC score of 0.74 (95% CI: 0.64–0.83), with the corresponding ROC curve shown in Figure 1. These results outperform similar studies that used LR for the same purpose. Although recent AI studies on SCD risk stratification typically achieved an AUC-ROC value in the 0.76–0.81 range, they used more complex models and, in most cases, images or ECG signal details. This suggests that the performance of AI models trained solely on routinely collected clinical data is comparable to more technically demanding approaches relying on advanced imaging or ECG processing. The 95% confidence interval of 0.64–0.83 indicates some uncertainty in the model’s predictive performance, suggesting that while the true AUC is likely to reflect at least moderate discriminatory ability, further validation on larger or independent datasets is needed to confirm the results.

### 3.2. Key Clinical Predictors

**Figure 2:**
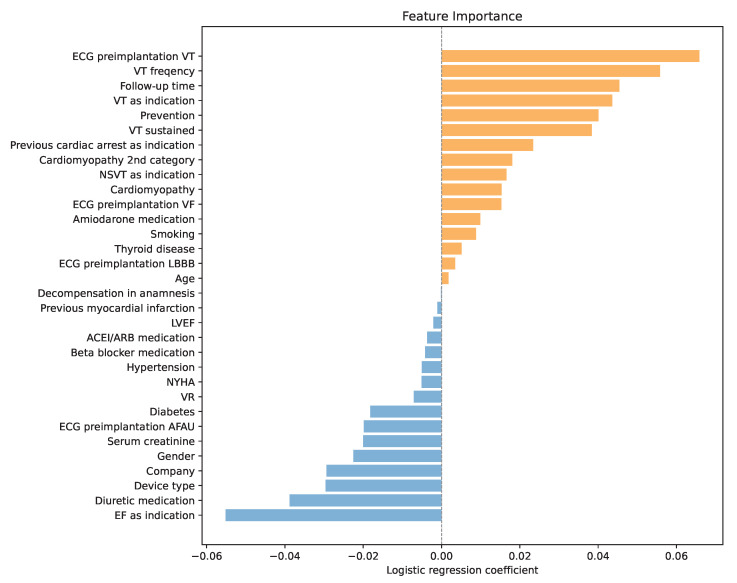
Variable importance through LR coefficients, orange for positive coefficients and blue for negative coefficients.

One of the main reasons for choosing LR as a predictor of high SCD risk was its interpretability. During training, each variable was assigned a LR coefficient, which represents the log of the odds ratio for that predictor. Positive coefficients increase the log-odds (and thus the odds) of the positive class, while negative coefficients decrease them. Predictors with larger absolute coefficients have a greater influence on the model’s predictions, with the sign indicating the direction of the effect. By analyzing the coefficients presented in Figure 2, a deeper understanding of variable importance and the model’s decision-making process was obtained.

The analysis confirmed that the history of ventricular tachycardia dominated the model, with higher frequencies increasing the risk of SCD even further. Another important factor was the follow-up time. A longer post-implantation period was associated with a higher probability of ICD activation. Similarly, sustained VT and secondary prevention emerged as significant risk enhancers. In addition, the analysis highlighted the prognostic contribution of renal function, with lower creatinine levels correlating with a higher probability of appropriate therapy, potentially reflecting the competing risk of non-arrhythmic death in patients with advanced renal dysfunction.

Interestingly, if the low ejection fraction was the primary indication for ICD implantation, the odds of an SCD were by 5 percentage points lower according to the LR. Another factor that the model recognized as important was the diuretic medication. Using diuretics reduced the model’s output, meaning it was less likely to predict an SCD. Moreover, the combination of furosemide and spironolactone reduced the chances of ICD even further. This observation may be linked to the established survival benefit of mineralocorticoid receptor antagonists in advanced heart failure and raises the possibility that optimized pharmacotherapy modifies arrhythmic risk profiles. In addition, female patients and patients with implanted CRT-D were less likely to suffer an SCD.

Like the standard clinical criteria, lower values of LVEF increased the risk of SCD. However, in the LR model, its influence was marginal.

### 3.3. Clinical Implications

Taken together, these results demonstrate that AI can extract clinically meaningful patterns from routine patient data. Rather than focusing on a single entry criterion such as LVEF, the models integrated multiple risk markers and generated more individualized risk profiles.

Importantly, this approach did not require advanced imaging or novel biomarkers but relied solely on data available in standard cardiology practice. This makes it potentially applicable across a wide range of healthcare settings, including those with limited access to advanced technology.

## 4. Discussion

The principal finding of this study is that AI, when applied to routinely collected clinical and device-related data, can meaningfully contribute to the identification of patients at risk of SCD. While ICDs remain the cornerstone of prevention, our results underscore a well-recognized limitation of current practice that guideline-based criteria, primarily driven by LVEF, lack the precision needed to separate those who will truly benefit from those who may never require device therapy [1,2,3,4,5,6,7,10].

Our analysis demonstrated that even LR, as a relatively simple ML model, achieved high sensitivity, correctly identifying most of the patients who eventually received appropriate ICD therapy. This variable is of particular clinical relevance. From a physician’s perspective, the gravest error is to miss a patient who is genuinely at risk. Even at the cost of lower specificity, the ability to capture almost all true positives suggests that ML could serve as an additional safeguard against undertreatment.

Key clinical predictors that emerged through our analysis are a history of ventricular tachycardia (frequency proportional with probability for SCD) and follow-up time (post-implantation period proportional with probability of ICD activation). Sustained VT and secondary prevention were identified as strong risk amplifiers. Better renal function (lower creatinine) was linked to higher rates of appropriate therapy. On the contrary, low ejection as the primary indicator for ICD implantation was associated with reduced odds of SCD. In addition, SCD prediction was less common in female patients and in those with implanted CRT-D devices. Furthermore, the use of diuretic medication turned out to reduce the output of the model, especially in the case of the combination of furosemide and spironolactone. When positioned within the wider body of literature, the deployed LR model trained on our dataset, achieving an AUC-ROC score of 0.74, delivers comparable performance to similar LR models reported in literature (0.65 reported in [24], 0.64 to 0.74 in [25,26,27,28,29] according to [15]). We acknowledge the existence of better-performing LR models, such as [30], according to [14] (up to 0.86), but achieved through incorporating a wider set of features, such as CMR.

Furthermore, we acknowledge the existence of other ML models reported in relevant literature that perform better (median AUC-ROC score 0.76 in a survey performed by Nazar et al. [14], 0.79 by Kolk et al. [31], and 0.80 by Barker et al. [15]). Nonetheless, these models are typically more complex and are developed using richer datasets, often incorporating ECG recordings or imaging-derived features. It is also worth mentioning that this increased complexity tends to reduce interpretability. Nevertheless, we confirmed that even a relatively simple model trained on routine data can perform at a level comparable to more complex ECG or imaging-based approaches. Incorporation of more advanced models, such as the ones used in those cases, will be done in future work.

Other groups have successfully applied AI to forecast inappropriate shocks, hospitalizations, and mortality [20,21], suggesting that the scope of clinical benefit could extend well beyond the decision of whether to implant a device. It is crucial to emphasize that our results should be interpreted as complementary to, not a replacement for, established clinical guidelines. All patients in our cohort were treated according to ESC recommendations, and ICD therapy remains a proven intervention for selected patients. However, the 2022 ESC guidelines explicitly recognize the shortcomings of relying on LVEF alone and acknowledge the role of additional markers such as myocardial fibrosis on cardiac MRI [10,11,12,13]. AI can be viewed as another layer in this evolving framework capable of synthesizing diverse clinical information into individualized risk estimates that reflect the complexity of real-world patients.

From a clinical perspective, the potential advantages of incorporating AI are clear. More precise stratification could reduce unnecessary device implantations and the complications associated with them, including lead failure, infection, and psychological burden. At the same time, patients with the greatest arrhythmic risk, particularly younger individuals and those with demonstrable arrhythmic substrates, could be identified with greater confidence and protected more effectively. Importantly, our approach required only data that are routinely available in most cardiology centers, enhancing its potential applicability across healthcare systems with varying levels of resources.

Several limitations warrant acknowledgment. This was a retrospective, single-center study, and external validation is needed before translation into practice. In addition, while our LR model demonstrated a high sensitivity of 86.50%, its modest precision (45.70%) renders it a not-yet-usable standalone decision tool. Also, although LR allows identification of independent associations, it does not establish causality. The interdependence among clinical variables and the complexity of SDC pathophysiology require careful interpretation supported by clinical expertise. Prospective, multicenter studies incorporating diverse populations and standardized protocols will be essential to confirm reproducibility and to evaluate whether AI-supported stratification leads to improved patient outcomes.

Direct head-to-head comparison with common heart failure scores was not performed due to an endpoint mismatch (overall mortality vs. appropriate ICD therapy) and incomplete availability of some required variables in this retrospective cohort. To avoid bias, we avoided partial implementations. A priority for future work is a prospective benchmarking framework, covering calibration, decision-curve, and reclassification analyses, to determine whether ML provides incremental clinical value beyond established scores while remaining aligned with guideline-directed care.

Future perspective and clinical integration. In current practice, ML should be positioned as an adjunct that supports, rather than replaces, guideline-directed therapy. Its value lies in synthesizing diverse clinical signals to aid, but never substitute individualized clinical judgment. A pragmatic path forward includes prospective multicenter validation, transparent model reporting (including calibration and explainability), and demonstration of real-world utility. If these steps consistently confirm benefit, ML-based risk estimates could be formalized as a structured scoring tool within future guideline frameworks while preserving individualized decision-making.

In summary, this study provides evidence that even a relatively simple ML model can extract clinically relevant signals from routine patient data and support more individualized decisions regarding ICD therapy. AI should be regarded as a promising adjunct to guideline-based criteria, with the potential to refine risk stratification and bridge the gap between population-based recommendations and the needs of individual patients. If validated in larger and more diverse cohorts, AI could become an integral part of the future strategy for SCD prevention.

## 5. Conclusions

In this study, we demonstrated that AI, applied to routine clinical and device-related data, can accurately identify patients most likely to benefit from ICD therapy. In our case, the LR model achieved high sensitivity, correctly recognizing most patients who eventually required life-saving intervention. These findings confirm that even a simple ML model is capable of reinforcing established predictors, uncovering new patterns, and generating more nuanced risk profiles than conventional criteria alone.

ML is expected to become a substantial aid to clinical decision-making, helping reduce non-beneficial implantations while safeguarding high-risk patients. Decisions must remain individualized, and, if validated in prospective studies, ML-based risk estimates could be incorporated as a formal scoring tool within future guidelines.

ML should be regarded as an adjunct to guideline-directed therapy rather than a substitute. Its incremental value over existing clinical scores should be confirmed through prospective, outcome-aligned comparisons before impacting practice or guideline adoption. Current European recommendations remain firmly grounded in clinical evidence, yet they acknowledge the limitations of an LVEF-based approach. By integrating diverse patient characteristics into individualized predictions, AI offers a pathway to refine existing frameworks and align them more closely with the complexity of real-world populations.

This technology offers a compelling clinical benefit. Enabling more efficient risk stratification can result in fewer unnecessary implantations, fewer complications, and a more timely protection for patients at the highest risk. At the same time, these results highlight the need for prospective, multicenter validation before routine adoption. Transparency, interpretability, and reproducibility will be critical to ensure safe translation into daily practice.

Ultimately, the value of AI lies not in replacing clinical judgment, but in augmenting it. By bridging the gap between population-based recommendations and individual patient realities, AI has the potential to move sudden cardiac death prevention toward a new era of precision medicine.

## Figures and Tables

**Figure A1:**
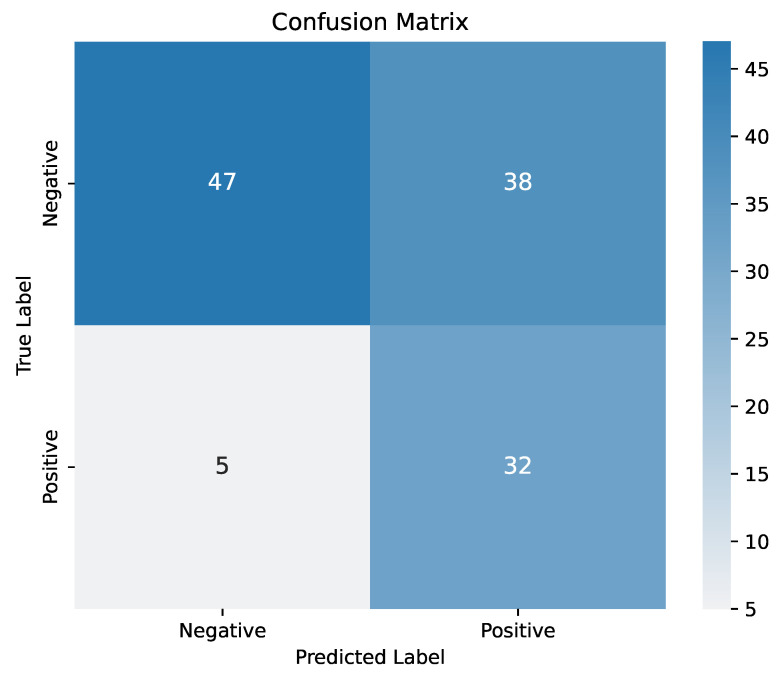
Confusion matrix.

## Data Availability

The data presented in this study are available on request from the corresponding author. The data are not publicly available due to ethical restrictions.

## Abbreviations

The following abbreviations are used in this manuscript:

ACEI	angiotensin-converting enzyme inhibitors
AF	Atrial fibrillation
AI	Artificial intelligence
ARB	Angiotensin receptor blockers
ARNI	Angiotensin receptor–neprilysin inhibitors
AUC-ROC	Area under the receiver operating characteristic curve
CMR	Cardiac magnetic resonance
CRT-D	Cardiac resynchronization therapy with defibrillator
ECG	Electrocardiogram
EF	Ejection fraction
ESC	European Society of Cardiology
ICD	Implantable cardioverter-defibrillator
LBBB	Left bundle branch block
LR	Logistic regression
LVEF	Left ventricular ejection fraction
MI	Myocardial infarction
ML	Machine learning
MRA	Mineralocorticoid receptor antagonists
NSVT	Non-sustained ventricular tachycardia
NYHA	New York Heart Association
SCD	Sudden cardiac death
VF	Ventricular fibrillation
VT	Ventricular tachycardia

## Appendix A. Variables

Table A1 and Table A2 present all variables used together with their distributions. For numerical variables, the mean and standard deviation are reported, whereas for categorical variables, the number and percentage of instances in the overall dataset are provided.

**Table A1:** Variables used in the study—part 1.

Variable	Statistics
Gender [N; male (%)]	506 (83.36%)
Age [years; M ± SD]	58.71 ± 13.32
Device type [N; ICD (%)]	472 (77.76%)
VR [N; VR (%)]	468 (77.10%)
Company [N; (%)]	
Medtronic	413 (68.04%)
St. Jude	42 (6.92%)
Biotronik	121 (19.93%)
Unknown	31 (5.11%)
Indication [N; yes (%)]	
Cardiac arrest	100 (16.47%)
Low EF	324 (53.38%)
VT	65 (10.71%)
NSVT	118 (19.44%)
Prevention [N; primary (%)]	457 (75.29%)
Cardiomyopathy [N; (%)]	
Ischemic	283 (46.62%)
Non-ischemic	196 (32.39%)
Dilatative	94 (15.49%)
General rhythm	15 (2.47%)
Non-compaction	9 (1.48%)
ARVD	5 (0.82%)
Toxic	4 (0.66%)
Thyrotoxic	1 (0.16%)
Cardiomyopathy 2nd cat [N; ishemic (%)]	283 (46.62%)
LVEF [%; M ± SD]	31.46 ± 11.58

**Table A2:** Variables used in the study—part 2.

Variable	Statistics
NYHA class [N; yes (%)]	
I	124 (20.43%)
II	300 (49.42%)
III	171 (28.17%)
IV	12 (1.98%)
VT sustained [N; yes (%)]	128 (21.09%)
VT frequency [Hz; M ± SD]	166.63 ± 41.93
ECG parameters [N; yes (%)]	
Ventricular tachycardia	369 (60.79%)
Ventricular fibrillation	82 (13.51%)
Atrial fibrillation/undulation	118 (30.97%)
Left bundle branch block	211 (34.76%)
Comorbidities [N; yes (%)]	
History of myocardial infarction	239 (39.37%)
History of decompensation	361 (59.47%)
Diabetes	165 (27.18%)
Hypertension	397 (65.40%)
Thyroid disease	106 (17.46%)
Serum creatinine [M ± SD]	111.41 ± 58.19
Smoking [N; yes (%)]	250 (41.19%)
Medication [N; yes (%)]	
Amiodarone	225 (37.07%)
ACEi/ARB	469 (77.27%)
Beta-blocker	559 (92.09%)
Diuretic [N; yes (%)]	169 (27.84%)
Furosemid + Spironolakton	262 (43.16%)
Follow-up period [months; M ± SD]	46.82 ± 24.67
Appropriate activation [N; yes (%)]	167 (27.51%)

## Appendix B. Confussion Matrix

Figure A1 shows the confusion matrix for the trained LR model evaluated on the test set. The test set comprised a randomly selected 20% of the total dataset, including 37 patients with appropriate ICD activations and 85 patients without. As shown in Figure A1, the model accurately identified most patients with appropriate ICD activation, correctly predicting 32 out of 37 cases. However, its performance was lower for patients without appropriate activation, misclassifying 38 of them as positive. This outcome was expected, as the LR model was trained to prioritize sensitivity and maximize the identification of true positive cases. Notably, considering that under the ESC guidelines, all patients would be classified as positive, correctly identifying 47 patients without appropriate activation represents a favorable improvement.

## References

[1] Moss A.J., Zareba W., Hall W.J., Klein H., Wilber D.J., Cannom D.S., Daubert J.P., Higgins S.L., Brown M.W., Andrews M.L. (2002). Prophylactic implantation of a defibrillator in patients with myocardial infarction and reduced ejection fraction. N. Engl. J. Med..

[2] Buxton A.E., Lee K.L., Fisher J.D., Josephson M.E., Prystowsky E.N., Hafley G. (1999). A randomized study of the prevention of sudden death in patients with coronary artery disease. N. Engl. J. Med..

[3] Bardy G.H., Lee K.L., Mark D.B., Poole J.E., Packer D.L., Boineau R., Domanski M., Troutman C., Anderson J., Johnson G. (2005). Amiodarone or an implantable cardioverter–defibrillator for congestive heart failure. N. Engl. J. Med..

[4] Kadish A., Dyer A., Daubert J.P., Quigg R., Estes N.M., Anderson K.P., Calkins H., Hoch D., Goldberger J., Shalaby A. (2004). Prophylactic defibrillator implantation in patients with nonischemic dilated cardiomyopathy. N. Engl. J. Med..

[5] Køber L., Thune J.J., Nielsen J.C., Haarbo J., Videbæk L., Korup E., Jensen G., Hildebrandt P., Steffensen F.H., Bruun N.E. (2016). Defibrillator implantation in patients with nonischemic systolic heart failure. N. Engl. J. Med..

[6] Elming M.B., Nielsen J.C., Haarbo J., Videbæk L., Korup E., Signorovitch J., Olesen L.L., Hildebrandt P., Steffensen F.H., Bruun N.E. (2017). Age and outcomes of primary prevention implantable cardioverter-defibrillators in patients with nonischemic systolic heart failure. Circulation.

[7] Akel T., Lafferty J. (2017). Implantable cardioverter defibrillators for primary prevention in patients with nonischemic cardiomyopathy: A systematic review and meta-analysis. Cardiovasc. Ther..

[8] Osman J., Tan S.C., Lee P.Y., Low T.Y., Jamal R. (2019). Sudden Cardiac Death (SCD)–risk stratification and prediction with molecular biomarkers. J. Biomed. Sci..

[9] Brown P.F., Miller C., Di Marco A., Schmitt M. (2019). Towards cardiac MRI based risk stratification in idiopathic dilated cardiomyopathy. Heart.

[10] Zeppenfeld K., Tfelt-Hansen J., Riva M.D., Winkel B.G., Behr E.R., Blom N.A., Charron P., Corrado D., Dagres N., Chillou C.D. (2022). 2022 ESC Guidelines for the management of patients with ventricular arrhythmias and the prevention of sudden cardiac death. Eur. Heart J..

[11] Freitas P., Ferreira A.M., Arteaga-Fernández E., de Oliveira Antunes M., Mesquita J., Abecasis J., Marques H., Saraiva C., Matos D.N., Rodrigues R. (2019). The amount of late gadolinium enhancement outperforms current guideline-recommended criteria in the identification of patients with hypertrophic cardiomyopathy at risk of sudden cardiac death. J. Cardiovasc. Magn. Reson..

[12] Di Marco A., Anguera I., Schmitt M., Klem I., Neilan T.G., White J.A., Sramko M., Masci P.G., Barison A., Mckenna P. (2017). Late gadolinium enhancement and the risk for ventricular arrhythmias or sudden death in dilated cardiomyopathy: Systematic review and meta-analysis. JACC Heart Fail..

[13] Pontremoli R., Borghi C., Perrone Filardi P. (2021). Renal protection in chronic heart failure: Focus on sacubitril/valsartan. Eur. Heart J.-Cardiovasc. Pharmacother..

[14] Nazar W., Szymanowicz S., Nazar K., Kaufmann D., Wabich E., Braun-Dullaeus R., Daniłowicz-Szymanowicz L. (2023). Artificial intelligence models in prediction of response to cardiac resynchronization therapy: A systematic review. Heart Fail. Rev..

[15] Barker J., Li X., Khavandi S., Koeckerling D., Mavilakandy A., Pepper C., Bountziouka V., Chen L., Kotb A., Antoun I. (2022). Machine learning in sudden cardiac death risk prediction: A systematic review. EP Eur..

[16] Kolk M.Z., Ruipérez-Campillo S., Alvarez-Florez L., Deb B., Bekkers E.J., Allaart C.P., Van Der Lingen A.L.C., Clopton P., Išgum I., Wilde A.A. (2024). Dynamic prediction of malignant ventricular arrhythmias using neural networks in patients with an implantable cardioverter-defibrillator. eBioMedicine.

[17] Kolk M.Z.H., Ruipérez-Campillo S., Deb B., Bekkers E.J., Allaart C.P., Rogers A.J., Van Der Lingen A.L.C.J., Alvarez Florez L., Isgum I., De Vos B.D. (2023). Optimizing patient selection for primary prevention implantable cardioverter-defibrillator implantation: Utilizing multimodal machine learning to assess risk of implantable cardioverter-defibrillator non-benefit. EP Eur..

[18] Jakaityte I., Brown S., Gillies K., Furniss G., Dayer M., Allen M. (2024). Machine learning can predict implantable cardioverter defibrillator therapy: A development study. Europace.

[19] Oberdier M.T., Neri L., Orro A., Carrick R.T., Nobile M.S., Jaipalli S., Khan M., Diciotti S., Borghi C., Halperin H.R. (2025). Sudden cardiac arrest prediction via deep learning electrocardiogram analysis. Eur. Heart J.-Digit. Health.

[20] Tateishi R., Suzuki M., Shimizu M., Shimada H., Tsunoda T., Miyazaki H., Misu Y., Yamakami Y., Yamaguchi M., Kato N. (2023). Risk prediction of inappropriate implantable cardioverter-defibrillator therapy using machine learning. Sci. Rep..

[21] Rosman L., Lampert R., Wang K., Gehi A.K., Dziura J., Salmoirago-Blotcher E., Brandt C., Sears S.F., Burg M. (2025). Machine learning-based prediction of death and hospitalization in patients with implantable cardioverter defibrillators. J. Am. Coll. Cardiol..

[22] Puljevic M., Ciglenecki E., Pasara V., Prepolec I., Dosen M.D., Hrabac P., Brekalo A.M., Bencic M.L., Krpan M., Matasic R. (2025). CRO-INSIGHT: Utilization of Implantable Cardioverter Defibrillators in Non-ischemic and Ischemic Cardiomyopathy in a Single Croatian Tertiary Hospital Centre. Rev. Cardiovasc. Med..

[23] Traykov V., Puererfellner H., Burri H., Foldesi C.L., Scherr D., Duncker D., Arbelo E., Botto G.L., Boriani G., Heidbuchel H. (2025). EHRA perspective on the digital data revolution in arrhythmia management: Insights from the association’s annual summit. Europace.

[24] Järvensivu-Koivunen M., Kallonen A., van Gils M., Lyytikäinen L.P., Tynkkynen J., Hernesniemi J. (2024). Predicting long-term risk of sudden cardiac death with automatic computer-interpretations of electrocardiogram. Front. Cardiovasc. Med..

[25] Vergara P., Tzou W.S., Tung R., Brombin C., Nonis A., Vaseghi M., Frankel D.S., Biase L.D., Tedrow U., Mathuria N. (2018). Predictive Score for Identifying Survival and Recurrence Risk Profiles in Patients Undergoing Ventricular Tachycardia Ablation. Circ. Arrhythmia Electrophysiol..

[26] Goldstein B.A., Chang T.I., Mitani A.A., Assimes T.L., Winkelmayer W.C. (2014). Near-Term Prediction of Sudden Cardiac Death in Older Hemodialysis Patients Using Electronic Health Records. Clin. J. Am. Soc. Nephrol..

[27] Shakibfar S., Krause O., Lund-Andersen C., Aranda A., Moll J., Andersen T.O., Svendsen J.H., Petersen H.H., Igel C. (2019). Predicting electrical storms by remote monitoring of implantable cardioverter-defibrillator patients using machine learning. EP Eur..

[28] Nakajima K., Nakata T., Doi T., Tada H., Maruyama K. (2022). Machine learning-based risk model using 123I-metaiodobenzylguanidine to differentially predict modes of cardiac death in heart failure. J. Nucl. Cardiol..

[29] Tse G., Zhou J., Lee S., Liu T., Bazoukis G., Mililis P., Wong I.C.K., Chen C., Xia Y., Kamakura T. (2020). Incorporating Latent Variables Using Nonnegative Matrix Factorization Improves Risk Stratification in Brugada Syndrome. J. Am. Heart Assoc..

[30] Bivona D.J., Tallavajhala S., Abdi M., Oomen P.J., Gao X., Malhotra R., Darby A.E., Monfredi O.J., Mangrum J.M., Mason P.K. (2022). Machine learning for multidimensional response and survival after cardiac resynchronization therapy using features from cardiac magnetic resonance. Heart Rhythm O2.

[31] Kolk M.Z., Ruipérez-Campillo S., Wilde A.A., Knops R.E., Narayan S.M., Tjong F.V. (2024). Prediction of sudden cardiac death using artificial intelligence: Current status and future directions. Heart Rhythm.

